# Supplementary figures and images for: Immunohistochemical study of histone protein 3 modification in pediatric osteosarcoma identifies reduced H3K27me3 as a marker of poor treatment response
Source: PLoS One. 2024 Nov 21;19(11):e0309471. doi: 10.1371/journal.pone.0309471 (PMC11581320; doi:10.1371/journal.pone.0309471)

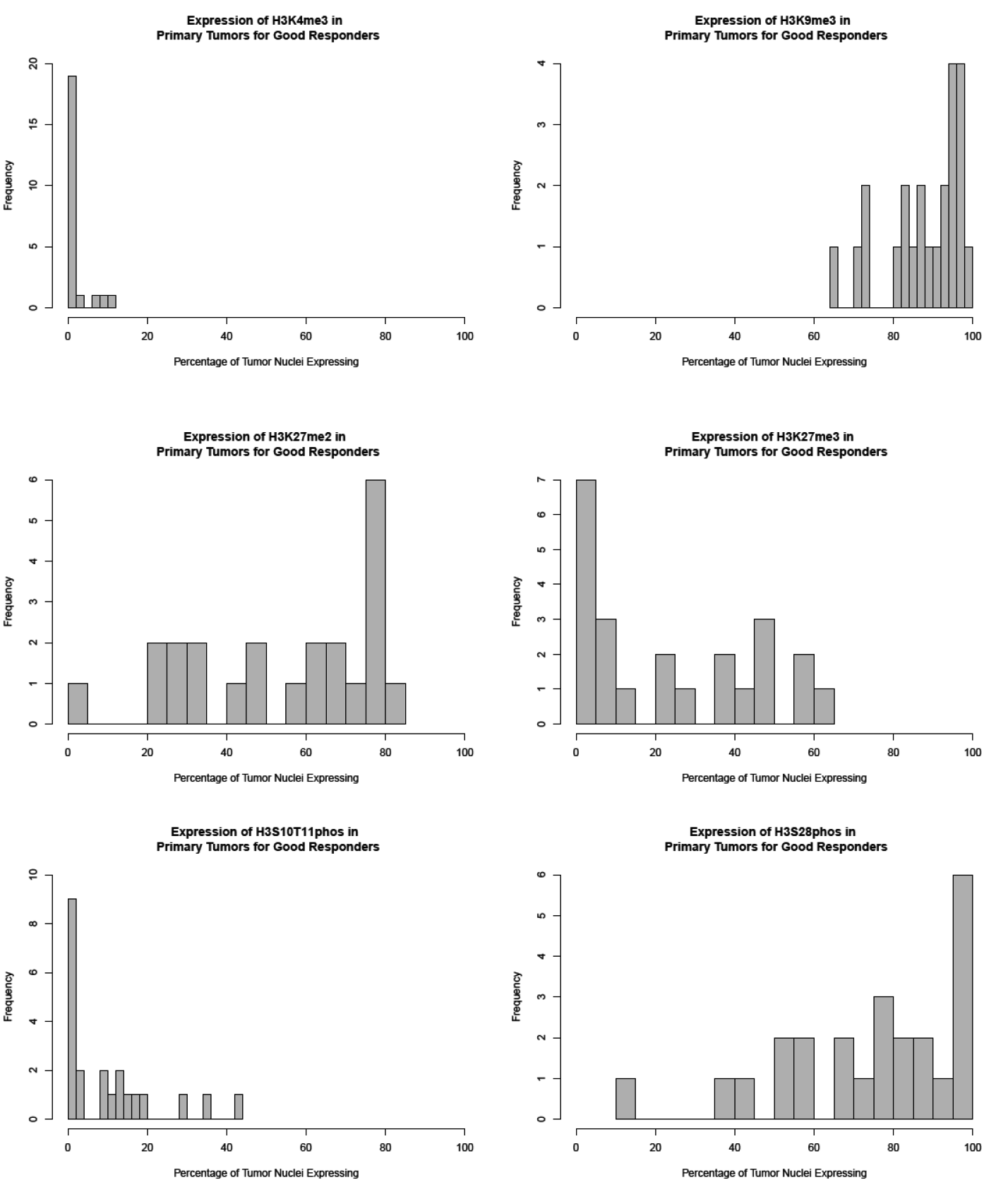

Supplement: S1 Fig — Histogram showing the distribution of IHC expression for good responders (≥90% tumor necrosis on resection). (TIF) [file pone.0309471.s001.tif]

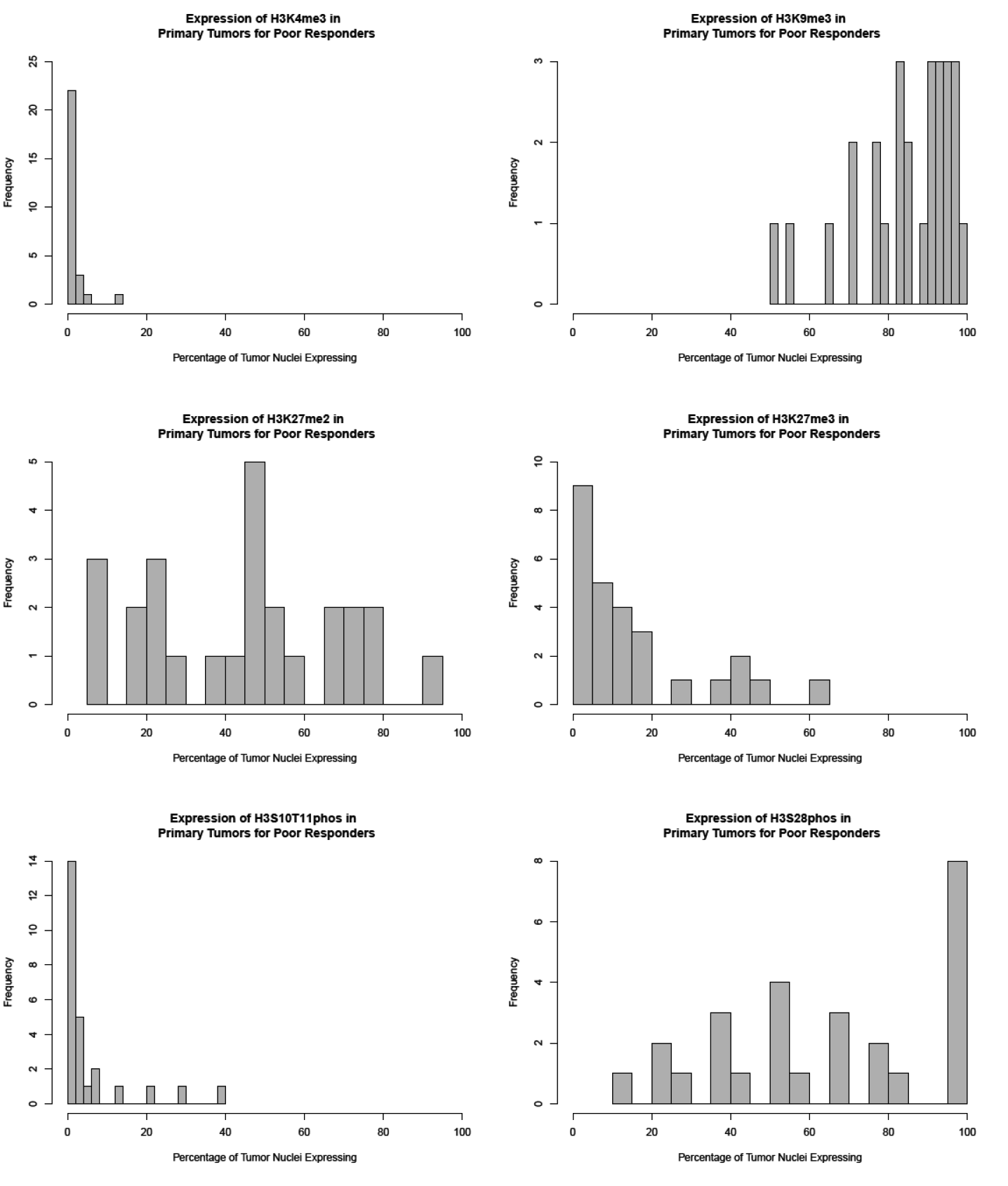

Supplement: S2 Fig — Histogram showing the distribution of IHC expression for poor responders (<90% tumor necrosis on resection). (TIF) [file pone.0309471.s002.tif]

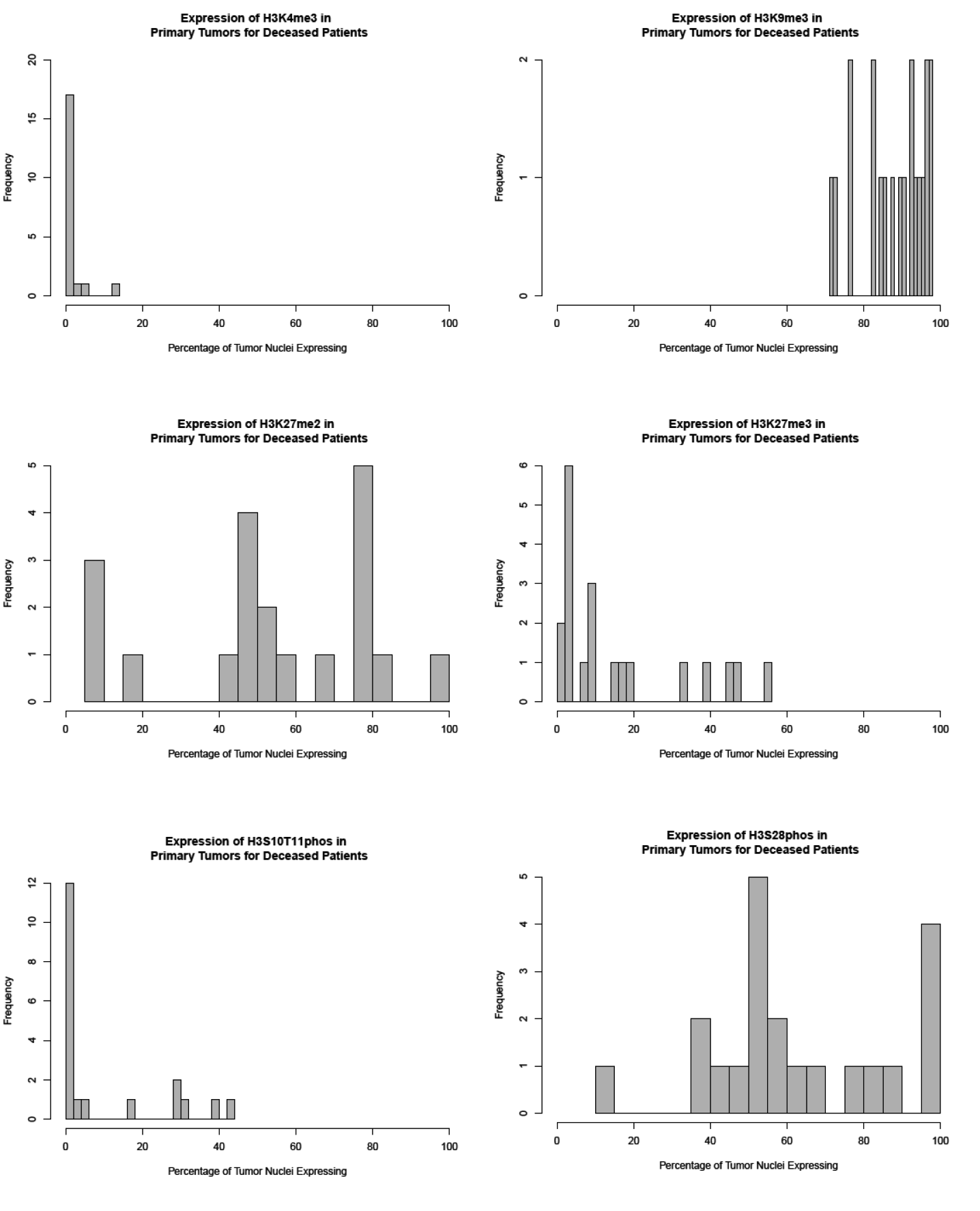

Supplement: S3 Fig — Histogram showing the distribution of IHC expression for patients and their primary tumor that died of their disease. (TIF) [file pone.0309471.s003.tif]

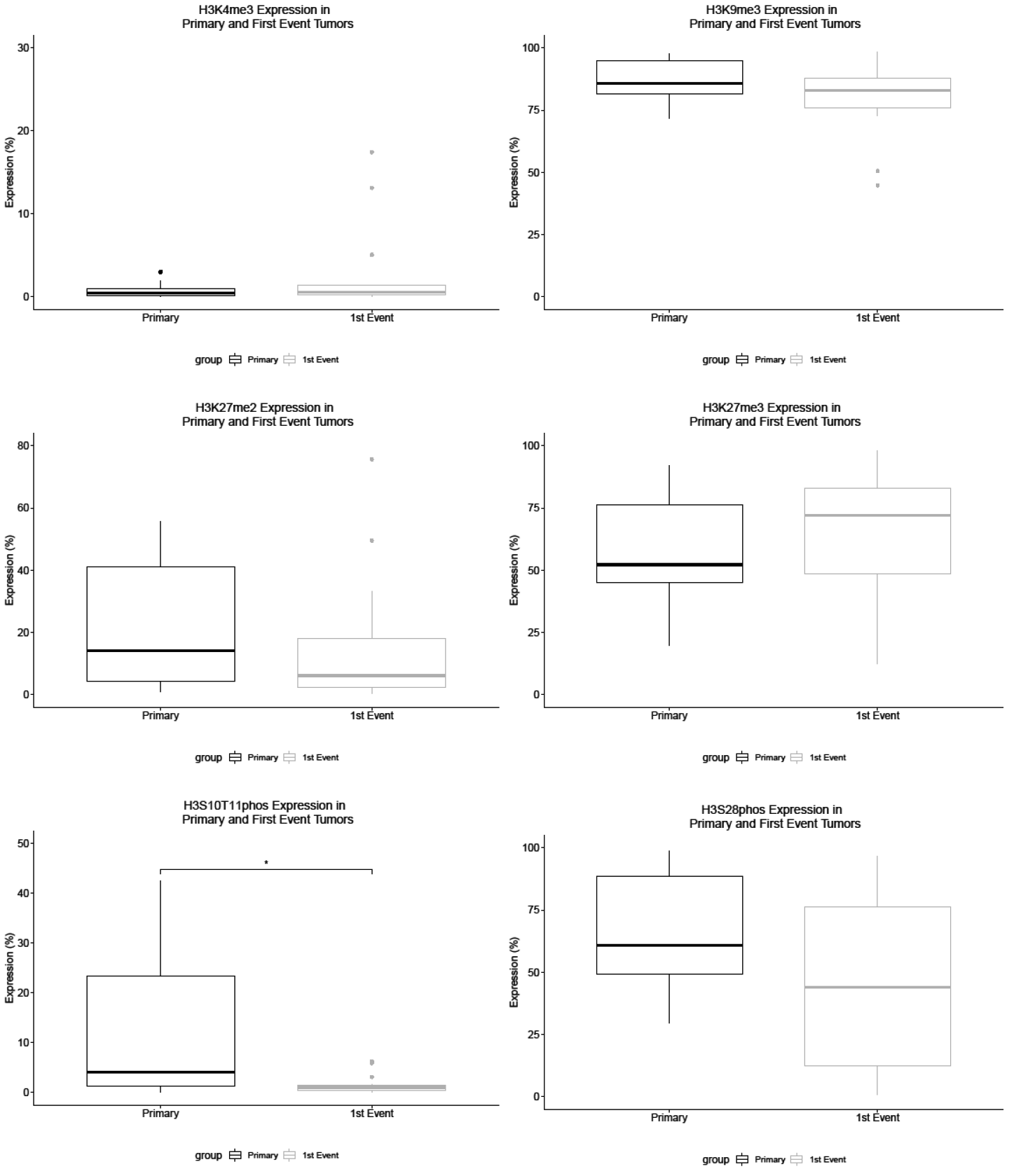

Supplement: S4 Fig — Box plot showing the expression of each IHC marker between the primary and 1st relapse. (TIF) [file pone.0309471.s004.tif]
